# Transcriptome Analysis Implicates Involvement of Long Noncoding RNAs in Cytoplasmic Male Sterility and Fertility Restoration in Cotton

**DOI:** 10.3390/ijms20225530

**Published:** 2019-11-06

**Authors:** Bingbing Zhang, Xuexian Zhang, Meng Zhang, Liping Guo, Tingxiang Qi, Hailin Wang, Huini Tang, Xiuqin Qiao, Kashif Shahzad, Chaozhu Xing, Jianyong Wu

**Affiliations:** State Key Laboratory of Cotton Biology, Institute of Cotton Research of Chinese Academy of Agricultural Science, Anyang 455000, Chinazhangxuexian@caas.cn (X.Z.); zhangmeng910305@163.com (M.Z.); guolp@cricaas.com.cn (L.G.); qitx@cricaas.com.cn (T.Q.); tanghn@cricaas.com.cn (H.T.); qiaoxq@cricaas.com.cn (X.Q.);

**Keywords:** long noncoding RNAs, anther development, CMS, fertility restoration, cotton

## Abstract

The cytoplasmic male sterility (CMS)/restorer-of-fertility system is an important tool to exploit heterosis during commercially hybrid seed production. The importance of long noncoding RNAs (lncRNAs) in plant development is recognized, but few analyses of lncRNAs during anther development of three-line hybrid cotton (CMS-D2 line A, maintainer line B, restorer-of-fertility line R) have been reported. Here, we performed transcriptome sequencing during anther development in three-line hybrid cotton. A total of 80,695 lncRNAs were identified, in which 43,347 and 44,739 lncRNAs were differentially expressed in A–B and A–R comparisons, respectively. These lncRNAs represent functional candidates involved in CMS and fertility restoration. GO analysis indicated that cellular hormone metabolic processes and oxidation–reduction reaction processes might be involved in CMS, and cellular component morphogenesis and small molecular biosynthetic processes might participate in fertility restoration. Additionally, 63 lncRNAs were identified as putative precursors of 35 miRNAs, and quantitative reverse transcription polymerase chain reaction (qRT-PCR) showed a similar expression pattern to RNA-seq data. Furthermore, construction of lncRNA regulatory networks indicated that several miRNA–lncRNA–mRNA networks might be involved in CMS and fertility restoration. Our findings provide systematic identification of lncRNAs during anther development and lays a solid foundation for the regulatory mechanisms and utilization in hybrid cotton breeding.

## 1. Introduction

Previous studies have shown that large portions of the eukaryotic genomic sequences consist of noncoding RNAs (ncRNAs). The ncRNAs lack apparent coding potential and could be divided into microRNAs (miRNAs) of 20–30 nt in length, medium ncRNAs of 50–200 nt, and long noncoding RNAs (lncRNAs) with transcripts longer than 200 nt [1,2,3,4]. The lncRNAs are a vital regulatory component of gene expression during many biological developmental processes [3,5,6]. In recent years, numerous lncRNAs have been identified in many plant species using RNA sequencing (RNA-seq) data. For example, Jun et al. identified 6480 long intergenic noncoding RNAs (lincRNAs) in Arabidopsis by analyzing transcriptome data [7]. In maize, 20,163 lncRNAs were identified in the complete genome using RNA-seq data [8]. In rice, 1624 lincRNAs and 600 lncNATs involved in sexual reproduction were identified using whole transcriptome RNA-seq [9].

Although many lncRNAs have been identified in diverse plant species, the functions for only a small number of lncRNAs have been elucidated. In Arabidopsis, the lncRNAs *COOLAIR* and *COLDAIR*, derived from the *FLOWERING LOCUS C* (*FLC*), are important in vernalization-mediated FLC repression [5,10,11]. In rice, a lncRNA termed long day-specific male fertility-associated RNA (LDMAR) may regulate photoperiod-sensitive male sterility (PSMS). Furthermore, a SNP between 58N and 58S in this lncRNA causes epigenetic modifications, which reduces expression of this lncRNA, resulting in male sterility under long-day conditions [12]. 

Additionally, many lncRNAs perform critical roles, with miRNAs by being their targets or precursors [13,14,15]. For example, lncRNAs in maize might act as precursors for miRNAs and function to regulate gene expression via an miRNA-dependent mechanism [13]. Cagirici et al. observed miRNA-related functions of lncRNAs and constructed miRNA-regulated networks between lncRNAs and mRNAs under drought stress in wheat [15]. Wang et al. analyzed the integrated expression of lncRNAs generating miR397 and their pivotal functions in regulating lignin metabolism in fibers of cotton [14]. In addition, target mimicry is a novel role for plant lncRNAs. For example, the lncRNA *IPS1* acts as a target mimic for miR399, which targets *PHO2* to control phosphate homeostasis in Arabidopsis [16,17,18]. Huang et al. predicted 15 lncRNAs as endogenous target mimics (eTMs) for 13 miRNAs in *Brassica*, of which two lncRNAs were shown to be functional eTMs for miR160 and to function in pollen development [19].

Cotton is one of the most important natural fibers utilized in the textile industry. In cotton, many lncRNAs involved in fiber development have been identified. Wang et al. identified 50,566 lincRNAs and 5826 long noncoding natural antisense transcripts (lncNATs) in *Gossypium barbadense* and reported that a lncRNA (*LINC02*) was highly expressed in lint-fuzz/linted-fuzzless cotton compared with lintless-fuzzless cotton [14]. Zou et al. systematically identified lncRNAs in cotton fibers and leaves of *Gossypium arboretum* [20]. Hu et al. identified 35,802 lncRNAs at the cotton fiber initiation stage and provided evidence for the potential functions of lncRNAs in fiber development by transcriptome sequencing, of which 645 and 651 lncRNAs were preferentially expressed in the fiberless mutant Xu-142-*fl* and fiber-attached lines, respectively [21]. In addition, Lu et al. analyzed the characteristics and expression patterns of lncRNAs under drought stress in cotton and concluded that lncRNAs may be involved in regulating plant hormone signaling pathways in response to drought stress [22]. Deng et al. developed a comprehensive catalogue of lncRNAs in Upland cotton under salt stress, of which 44 lincRNAs were differentially expressed under salt stress, and these lincRNAs may target mRNAs via *cis*-acting regulation [23]. However, lncRNAs involved in anther development in three-line hybrid cotton have not been identified, although transcriptomic analysis during anther development has been undertaken [24,25]. Here, we present a detailed analysis of anther development-related lncRNAs and mRNAs and their specific interactions in three-line hybrid cotton carrying cytoplasmic male sterile *Gossypium harknessii* (CMS-D2) cytoplasm. 

## 2. Results

### 2.1. Genome-Wide Identification and Characterization of LncRNAs during Anther Development of Three-Line Hybrid Cotton

**Figure 1:**
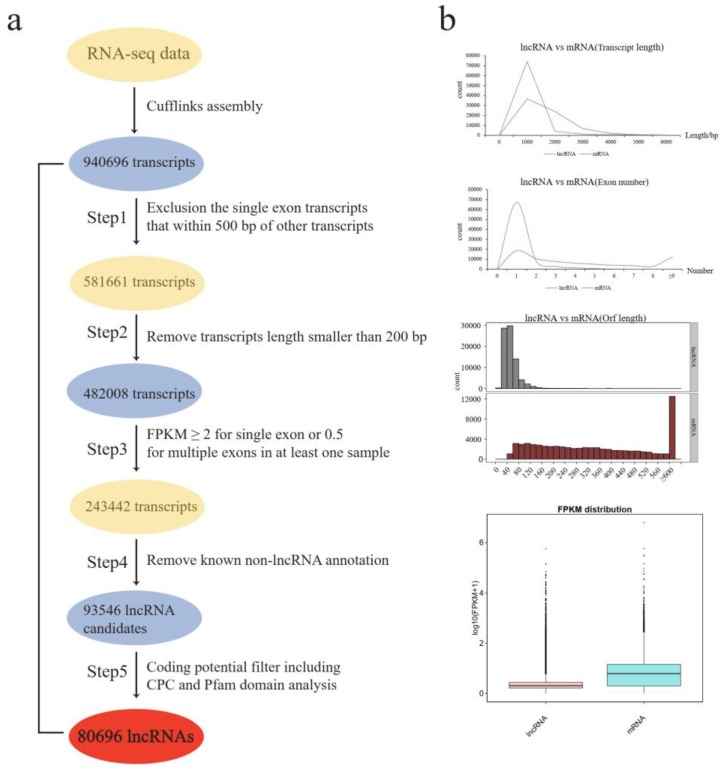
Identification and characterization of long noncoding RNAs (lncRNAs) in *Gossypium hirsutum*. (**a**) The pipeline for the identification of lncRNAs in *Gossypium hirsutum*; (**b**) Compare the characteristics of lncRNAs and mRNAs in *Gossypium hirsutum*.

To identify the lncRNAs involved in cytoplasmic male sterility (CMS) and restoration of fertility during anther development, high-throughput sequencing was performed for the CMS line (A), maintainer line (B), and fertility restoration line (R), each with three biological replicates. In total, 910 million clean reads (284 million in A, 315 million in B, and 311 million in R) passed the quality filters and were retained for further analysis. Almost 86%, 87%, and 88% of the reads were aligned to the TM-1 reference genome for A, B, and R, respectively (Table 1). On the basis of the transcripts assembly, a total of 940,696 transcripts were identified. To identify putative lncRNAs, we first filtered out the single-exon transcripts located within 500 bp of other transcripts, then excluded short transcripts (length < 200 bp), and screened the transcripts based on expression level (FPKM ≥ 0.5 for multi-exon transcripts, or FPKM ≥ 2 for single-exon transcripts), and finally filtered out the known non-lncRNAs. After these basic filtering processes, 93,546 transcripts were retained as lncRNA candidates. We evaluated the coding potential of the remaining transcripts by means of CPC (coding potential calculator) and Pfam protein domain analyses (Figure 1a). After the five-step filter process, 80,695 lncRNAs were identified during anther development (Table S1).

To describe explicitly the characteristics of lncRNAs, we compared the characteristics of lncRNAs and mRNAs in the following aspects (Figure 1b). In transcript length, the lncRNAs length ranged from 200 to 18,412 bp, and more than 97% of the lncRNAs were smaller than 2000 bp in length, whereas the length of mRNAs ranged from 150 to 21,501 bp, but almost 85% of mRNAs were between 150 and 2000 bp in length. In exon number, lncRNAs had fewer exons than mRNAs on average, almost 83% of the lncRNAs contained one exon, and 17% had multiple exons, whereas almost 26% of the mRNAs contained one exon and 74% had multiple exons. The overall open reading frame (ORF) length of lncRNAs was typically smaller than that of mRNAs. Comparison of FPKM distribution between lncRNAs and mRNAs showed that the FPKM of lncRNAs was lower than that of mRNAs during anther development (Table S1). 

### 2.2. Identification and Functional Analysis of Differentially Expressed LncRNAs in A, B, and R Lines

**Figure 2:**
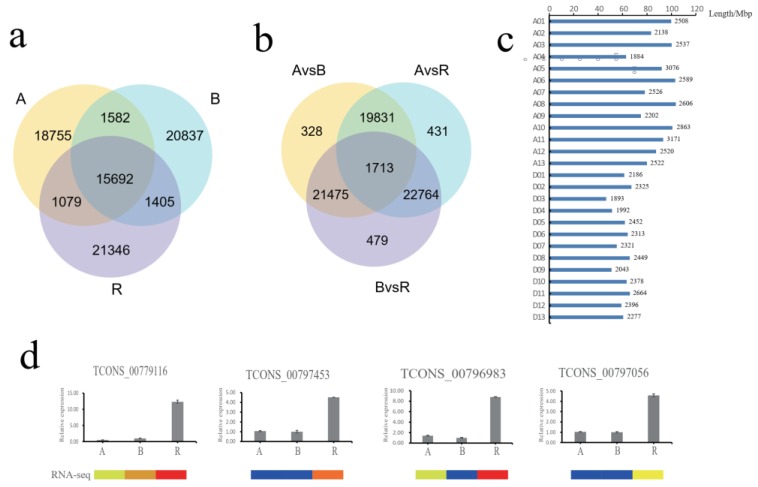
Venn diagram showing the common and distinct lncRNAs expression in A, B, and R lines. (**a**) The common and distinct lncRNAs identified in A, B, and R lines; (**b**) The common and distinct differentially expressed lncRNAs identified in A–B, A–R, and B–R comparison. (**c**) The distribution of differentially expressed lncRNAs among the A and D subgenome of *G. hirsutum*. (**d**) The analysis of qRT-PCR with differentially expressed lncRNAs identified in the *Rf1* region of Gh_D05 chromosome.

The following three comparisons of lncRNA expression levels were performed: A–B, which had the isogenic nuclear genomes (containing the recessive non-functional *rf1* allele) but different cytoplasm and fertility; A–R, both of which had the same CMS-D2 cytoplasm but differed in fertility and *Rf1* alleles; and B–R, both of which were isogenic and fertile but differed in cytoplasm and *Rf1* alleles. A total of 18,755, 20,837, and 21,346 unique lncRNAs were specifically expressed in the A, B, and R lines, respectively, and 15,692 lncRNAs were expressed in common among the A, B, and R lines (Figure 2a). On the basis of the expression level, lncRNAs with a greater than two-fold change and *p*-value < 0.01 were considered to be differentially expressed in different samples. A total of 43,347, 44,739, and 46,431 differentially expressed lncRNAs were identified in the A–B, A–R, and B–R comparisons, respectively, and 1713 lncRNAs were differentially expressed in common among the A–B, A–R, and B–R comparisons (Figure 2b, Table S2). The differentially expressed lncRNAs represented a total of 67,021 non-redundant lncRNAs that were distributed among the 26 chromosomes of *G. hirsutum*, of which 33,142, 29,689, and 4190 differentially expressed lncRNAs were located in the A and D subgenomes and different scaffolds, respectively (Table S2). The distribution of differentially expressed lncRNAs for the three comparisons is shown in Figure 2c. 

Previous studies indicate that the *Rf1* gene is located on chromosome Gh_D05, and the nearest flanking simple sequence repeat (SSR) markers to *Rf1* are BNL3535 at a genetic distance of 0.049 cM and NAU3652 with a genetic distance of 0.078 cM [26,27]. In the present study, a total of 2452 differentially expressed lncRNAs were identified on chromosome Gh_D05, of which 806 lncRNAs were differentially expressed in the A–R group. Furthermore, 86 lncRNAs were identified in the *Rf1* region flanked by the two SSR markers, of which 65 lncRNAs were down-regulated and 21 lncRNAs were up-regulated in the R line compared with the A and B lines. For example, the RNA-seq data showed that *TCONS_00779116*, *TCONS_00797453*, *TCONS_00796983*, and *TCONS_00797056* were up-regulated in the fertile R line, compared with the A and B lines, and the qRT-PCR results were approximately consistent (Figure 2d). 

**Figure 3:**
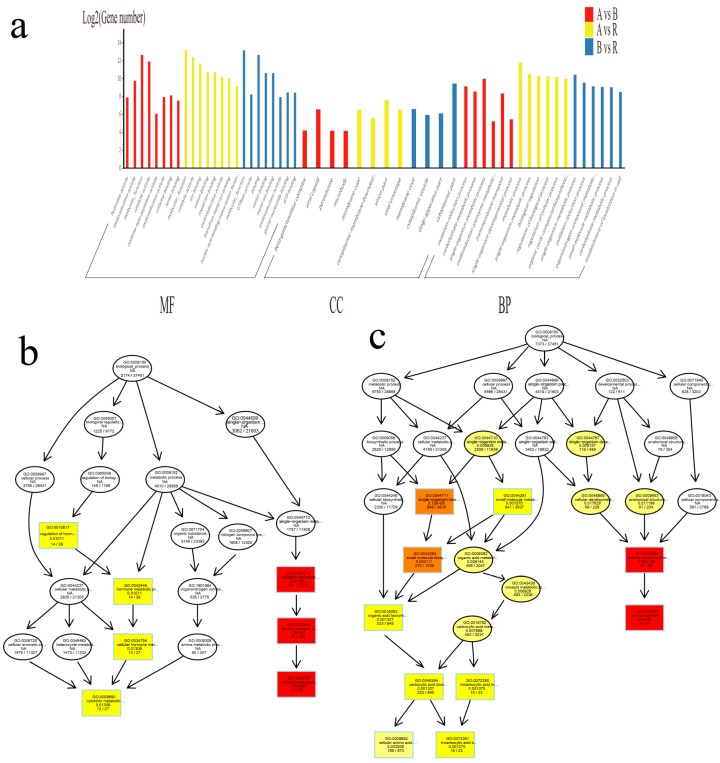
Functional analysis of differentially expressed lncRNAs in A, B and R lines. (**a**) Gene ontology (GO) analysis of genes regulated by differentially expressed lncRNAs in A, B and R lines; (**b**) GO enrichment analysis of genes regulated by differentially expressed lncRNAs in A–B comparison; (**c**) GO enrichment analysis of genes regulated by differentially expressed lncRNAs in A–R comparison.

It has been shown that lncRNAs are preferentially located close to genes that they regulate, and that lncRNAs might overlap with the promoter region and may regulate the expression profile of their target genes at the transcriptional or post-transcriptional level [6,17,28,29,30]. In the present study, to evaluate the function of these differentially expressed lncRNAs, we selected the genes located less than 10 kb from the differentially expressed lncRNAs as corresponding target genes and performed GO analyses among the A–B, A–R, and B–R comparisons (Figure 3a, Table S3). The GO terms oxidation–reduction process, regulation of hormone levels, and hormone metabolic process were the three most highly enriched terms in the A–B comparisons, whereas cell morphogenesis, cellular component morphogenesis, and the oxidation–reduction process were the three most enriched terms in the A–R comparisons. Similarly, in B–R comparisons, the three most enriched terms were cell morphogenesis, cellular component morphogenesis, and the single-organism biosynthetic process.

To identify lncRNAs associated with CMS or fertility restoration, differentially expressed lncRNAs between A–B and A–R comparisons were analyzed. In total, 43,347 and 44,739 differentially expressed lncRNAs were identified in the A–B and A–R comparison groups, respectively. Of these differentially expressed lncRNAs, 328 and 431 were unique in the A–B and A–R comparisons and thus might be involved in CMS or fertility restoration during anther development of cotton. In addition, we detected significant enrichment of GO terms involved in CMS or fertility restoration (*p* < 0.05). In the A–B comparison, we observed GO term enrichment for biological processes, including oxidation–reduction process (GO: 0055114), photosynthetic electron transport chain (GO: 0009767), regulation of hormone levels (GO: 0010817), cellular hormone metabolic process (GO: 0034754), and cytokinin metabolic process (GO: 0009690) (Figure 3b). These results showed that the greatest difference between normal Upland cotton cytoplasm and sterile cytoplasm was enrichment in the cellular hormone metabolic process and oxidation–reduction reaction process. In the A–R comparison, the most significantly enriched GO terms among biological processes were cellular component morphogenesis (GO: 0032989) and small molecular biosynthetic process (GO: 0044283), of which cellular amino acid biosynthetic process (GO: 0008652) and tricarboxylic acid biosynthetic process showed significant enrichment (GO: 0072351) (Figure 3c, Table S3). These results indicated that differentially expressed lncRNAs may regulate functional genes involved in cellular component morphogenesis for fertility restoration in cotton.

### 2.3. Predicted Interactions between LncRNAs and MiRNAs during Anther Development

**Figure 4:**
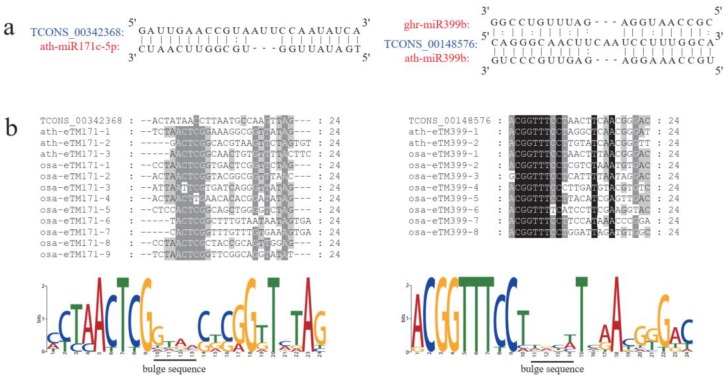
LncRNAs acting as endogenous target mimics (eTMs) of miRNAs in anther development of cotton. (**a**) The prediction lncRNAs as eTMs of ath-miR171c-5p and ath-miR399b in cotton; (**b**) Sequence alignment of eTMs for ath-miR171c-5p and ath-miR399b in cotton, Arabidopsis, and rice.

MiRNAs regulate gene expression at the post-transcriptional level by interacting with the complementary binding sites on target sequences, resulting in mRNA cleavage, decoy activity, and translation repression [15,31]. A previous study indicated that lncRNAs may act as targets or eTMs by binding with miRNAs and thus inhibit the interaction between miRNAs and the target genes [18]. In the current study, we predicted the potential of lncRNAs as miRNA eTMs by integration of previous miRNA sequence data during anther development [25]. In total, two lncRNAs (*TCONS_00342368* and *TCONS_00148576*) were predicted to be potential eTMs for five miRNAs, of which *TCONS_00342368* was a putative eTM for ath-miR171c-5p, osa-miR171c-5p, and stu-miR171d-5p, and *TCONS_00148576* was a putative eTM for ath-miR399b and cme-miR399d (Figure 4a). The predicted miRNA binding sites and the bulge region in lncRNAs were identical among the different miRNAs in the same miRNA family. To analyze the sequence evolutionary conservation of these eTM lncRNAs, we aligned the sequences of the predicted eTM-binding sites for miR171 and miR399 from Arabidopsis and rice (Figure 4b). The miRNA binding sequences were well conserved, and the bulge region frequently varied among different species, consistent with previous studies of Arabidopsis and rice [18] and *Brassica* [19]. Further analysis is necessary to understand the role of the two lncRNAs predicted to be eTMs.

**Figure 5:**
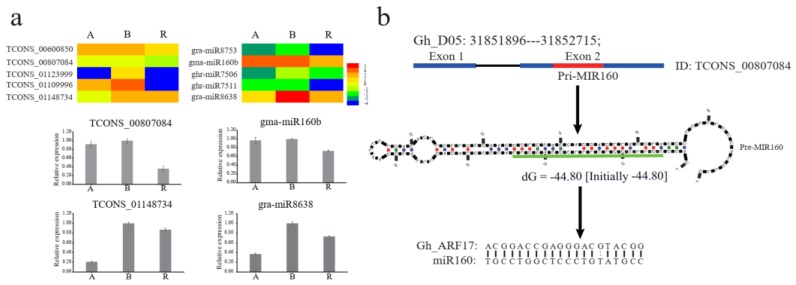
Expression and functional prediction of lncRNAs generating corresponding miRNAs. (**a**) RNA-seq and qRT-PCR validate the expression level of miRNAs precursor lncRNAs; (**b**) The module of lncRNAs generating corresponding gma-miR160b to regulate target gene. The red line in exon 2 is the miR160 precursor. The green line is the mature sequence of miR160.

In addition, lncRNAs might have functions associated with a role as miRNA precursors [32]. Those lncRNAs that act as precursors of miRNAs might perform an indirect regulatory function through corresponding miRNAs. Moreover, differential expression of lncRNAs might result in the differential expression of corresponding mature miRNAs [15,32]. In the present study, 63 lncRNAs were predicted to be putative precursors of 35 miRNAs belonging to 26 miRNA families (Table S4). Of these lncRNAs, 13 lncRNAs were identified as the putative precursor of multiple miRNAs, with 1–2 nt difference in the mature miRNA sequence. Interestingly, 19 of the precursor lncRNAs showed differential expression among the A, B, and R lines, of which five lncRNAs (*TCONS_00600850*, *TCONS_00807084*, *TCONS_01123999*, *TCONS_01109996*, and *TCONS_01148734*) showed a consistent expression pattern with the corresponding mature miRNA (gra-miR8753, gma-miR160b, ghr-miR7506, ghr-miR7511, and gra-miR8638). The results of qRT-PCR analysis showed that two lncRNAs (*TCONS_00807084* and *TCONS_01148734*) and the corresponding miRNAs (gma-miR160b and gra-miR8638) showed a similar expression pattern in three-line hybrid cotton (Figure 5a). Interestingly, gma-miR160b, which is derived from *TCONS_00807084*, was up-regulated in the A and B lines compared with the R line and may regulate an auxin response factor *GhARF17* (*Gh_D06G0360*) (Figure 5b). Thus, *TCONS_00807084* and gma-miR160b might play critical roles in anther development by influencing the auxin regulatory pathway. These findings indicate the complex regulatory mechanisms by which lncRNAs and the corresponding mature miRNAs function during anther development, although the underlying regulatory network remains unclear.

### 2.4. The miRNA–LncRNA–mRNA Regulatory Networks between A, B, and R Lines

**Figure 6:**
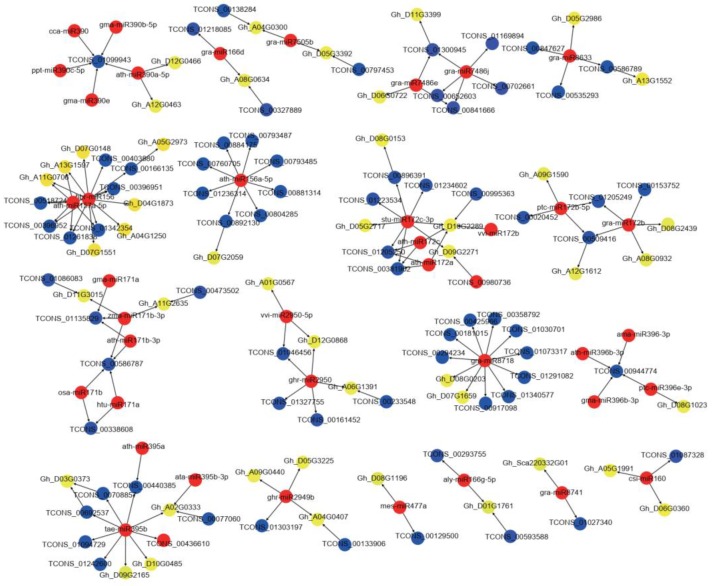
The representative miRNAs-lncRNAs-mRNAs regulatory networks in anther development of cotton. Red nodes: miRNAs; Blue nodes: lncRNAs; Yellow nodes: mRNAs. Arrow direction means regulator to targets.

To explore the regulatory networks of lncRNAs involved in CMS and fertility restoration, we selected the target genes of the differentially expressed lncRNAs and miRNAs based on the RNA-seq data and constructed a putative miRNA–lncRNA–mRNA regulatory network using Cytoscape software (Figure 6, Table S5). The networks were composed of 66 miRNAs, 161 lncRNAs, and 658 mRNAs (mRNAs regulated by miRNAs and lncRNAs), of which 58 lncRNAs regulated by 15 miRNAs and 77 lncRNAs regulated by 35 miRNAs were specifically differentially expressed in the A–B and A–R comparisons, respectively; in addition, 26 lncRNAs regulated by 16 miRNAs were in common among the A–B and A–R comparisons. These predicted target genes regulated by miRNAs and lncRNAs were divided into multiple groups. First, several genes showed critical roles in oxidation–reduction processes, of which a glutamyl-tRNA reductase (*Gh_A08G0634*), cupredoxin superfamily protein (*Gh_D03G0412*), and cinnamyl alcohol dehydrogenase (*Gh_D11G3399*) show oxidoreductase activity during anther development. Second, several genes were transcription factors and involved in cellular hormone response and metabolic processes. For example, bZIP (*Gh_A01G1768*), ARF (*Gh_D06G0360*), and SPL (*Gh_A11G0706*) transcription factors and SAUR-like auxin-responsive protein (Gh_A12G2237) and a gibberellin-regulated family protein (Gh_D02G0666) were regulated by miRNAs and lncRNAs involved in hormone response and metabolic processes. Third, several genes were functional proteins, including an ABORTED MICROSPORES (AMS) protein (Gh_D12G0328), nucleotide/sugar transporter family protein (Gh_A04G0407), and NB-ARC domain-containing disease resistance protein (Gh_A08G1378). Several functionally unknown genes regulated by miRNAs and lncRNAs were differentially expressed in the A, B, and R lines. These above-mentioned genes may play critical roles in CMS and fertility restoration in cotton.

**Figure 7:**
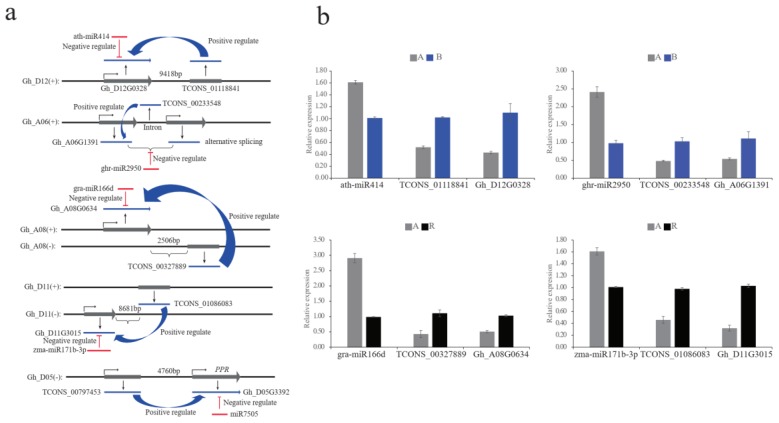
The putative regulation mechanism and expression pattern analysis in miRNAs–lncRNAs–mRNAs networks during anther development. (**a**) Regulation mechanism prediction of functional miRNAs–lncRNAs–mRNAs networks in anther development; (**b**) The qRT-PCR validate four miRNAs–lncRNAs–mRNAs regulatory networks in anther development of cotton.

The expression levels of several miRNA–lncRNA–mRNA regulatory networks were validated by qRT-PCR and were concordant with the transcriptome data (Figure 7). For example, a bHLH transcription factor, ABORTED MICROSPORES (AMS) gene (*Gh_D12G0328*), was a specific phytochrome-interacting factor regulated by ath-miR414 and TCONS_01118841. The gene *Gh_A06G1391*, which was regulated by ghr-miR2950 and TCONS_00233548, shows oxidoreductase activity and may participate in fatty acid biosynthesis. The qRT-PCR results showed that both genes were down-regulated in the A line compared with the B line. These genes might be involved in CMS during anther development. In the A–R comparison, a glutamyl-tRNA reductase (*Gh_A08G0634*) functions in oxidation–reduction processes, as the target gene of gra-miR166d and TCONS_00327889 and was down-regulated in the A line compared with the R line. The gene *Gh_D11G3015*, which encodes a calcium-dependent lipid-binding (CaLB domain) protein and was the target gene of zma-miR171b-3p and TCONS_01086083, may participate in the calcium signaling pathway and was down-regulated in the A line compared with the R line. These genes might be involved in pollen development and fertility restoration during anther development. In addition, in our previous study, the *PPR* (*Gh_D05G3392*) gene located near the region of *Rf1* was regulated by gra-miR7505b [25]. Interestingly, we detected the lncRNA *TCONS_00797453* located in the promoter region of the *Gh_D05G3392* gene and may strongly regulate the expression of that gene in the R line. This result showed that *PPR* (*Gh_D05G3392*) may be regulated by both gra-miR7505b and *TCONS_00797453* to participate in fertility restoration.

## 3. Discussion

Although an increasing number of lncRNAs have been identified in cotton, including those involved in fiber development [14,20,21], response to drought and salt stress [22,23], and disease resistance [33], no lncRNAs have been previously identified during anther development in three-line hybrid cotton. In the present study, transcriptome sequencing was performed during anther development (at the stage of male meiosis) in Upland cotton harboring the CMS-D2 cytoplasm to systematically identify lncRNAs involved in CMS and fertility restoration. The differences between the CMS line (A), maintainer line (B), and fertility restoration line (R) are as follows: A vs. B, both have different cytoplasm and fertility; A vs. R, both harbor different *Rf1* alleles and fertility; and B vs. R, both differ in cytoplasm and *Rf1* alleles. Thus, three-line hybrid cotton represents suitable material to explore the molecular mechanism of nucleo-cytoplasmic interaction. In our previous studies, differentially expressed genes and miRNAs were analyzed during anther development in three-line hybrid cotton, and many candidate genes and miRNAs were discovered [24,25]. In the current study, identification of lncRNAs differentially expressed between the A, B, and R lines provides a novel perspective for understanding the molecular mechanism of CMS and fertility restoration in Upland cotton. 

### 3.1. Overview of LncRNAs Identification and Function in Anther Development 

In the present study, 80,695 lncRNAs were identified by analyzing almost 910 million clean reads, of which 18,755, 20,837, and 21,346 unique lncRNAs were specifically expressed in the A, B, and R lines, respectively. As previously reported [14], we observed that the number of identified lncRNAs during anther development is larger than the numbers identified in Arabidopsis, maize, and *Gossypium arboreum* [7,8,20] but similar to the numbers identified in wheat [15], *Brassica rapa* [19], and cotton in response to drought stress [22]. Thus, we suspect that the size and complexity of the genome and strict screening criteria may have led to an increase in the number of lncRNAs identified. In addition, comparison of the characteristics of lncRNAs and mRNAs revealed that lncRNAs share many common characteristics, such as fewer exons, typically smaller length, and lower expression level than mRNAs during anther development. 

Few studies have investigated the involvement of lncRNAs in plant reproductive development. In rice, Zhang et al. identified and verified a set of lncRNAs involved in sexual reproduction [9]. Huang et al. identified lncRNAs during pollen development and fertilization in *Brassica*. The GO enrichment analysis indicated that genes that show transcription regulator activity (GO: 0030528) and involved in morphogenesis (GO: 0010927) might perform critical roles in pollen exine formation [19]. In the current study, 43,347 and 44,739 lncRNAs were differentially expressed in the A–B and A–R comparisons, respectively. Previous studies indicate that *Rf1* is located on chromosome Gh_D05 and is flanked by the BNL3535 and NAU3652 SSR markers [26,27]. In the present study, 21 lncRNAs in the *Rf1* region flanked by these two markers were up-regulated in the R line compared with the A and B lines, and the results of qRT-PCR analysis were approximately consistent with the RNA-seq data. 

To explore the function of the differentially expressed lncRNAs, we performed a GO enrichment analysis. In the A–B comparison, enrichment was observed in the GO terms oxidation–reduction process, photosynthetic electron transport chain, and cellular hormone regulation and metabolic process. These results indicated that the differences between the normal Upland cotton cytoplasm and sterile cytoplasm may influence cellular hormone and metabolic processes and oxidation–reduction reaction processes. Energy supply and cellular hormone content and metabolism during anther development might be involved in CMS. In the A–R comparison, the most significantly enriched GO terms were cellular amino acid biosynthetic process and tricarboxylic acid biosynthetic process. These results indicated that differentially expressed lncRNAs may participate in cellular component morphogenesis and small molecular biosynthetic processes for fertility restoration in cotton. These lncRNAs represent functional candidates for CMS and fertility restoration for further investigation.

### 3.2. Relationship between LncRNAs and MiRNAs in Anther Development of Cotton

Previous studies indicate that lncRNAs may act as eTMs to prevent interaction between miRNAs and the target genes by competitively binding with the corresponding miRNAs [16,18,19]. For example, the lncRNA *IPS1* in Arabidopsis acts as an eTM of ath-miR399 to regulate *PHOS2* and a 3 nt bulge is present in the 10th and 11th nt positions of the miRNA [16]. In *Brassica*, two lncRNAs are eTMs of miR160 and function in pollen formation and male fertility [19]. In tomato, a lncRNA acts as an eTM of miR166 and may regulate Tomato yellow leaf curl virus resistance [34]. In the present study, two lncRNAs (*TCONS_00342368* and *TCONS_00148576*) were predicted to be potential eTMs for five miRNAs, of which *TCONS_00342368* was a putative eTM for ath-miR171c-5p, osa-miR171c-5p, and stu-miR171d-5p, and *TCONS_00148576* was a putative eTM for ath-miR399b and cme-miR399d. The predicted miRNA binding sites and the bulge region are conserved among different miRNAs and the same miRNAs in different plant species [18,19,35]. We observed that the sequence of the eTMs of miR171 and miR399 were well conserved and the bulge region frequently varied among different species, which is consistent with previous studies [18,19,35]. Therefore, we hypothesize that certain interactions between these lncRNAs and miRNAs may play a fundamental role in anther development of cotton.

In addition to functioning as eTMs of miRNAs, lncRNAs are also predicted to be precursors of miRNAs and differential expression of lncRNAs might result in the differential expression of the corresponding mature miRNAs [13,14,15]. For instance, Wang et al. systematically analyzed the expression of a lncRNA that generates miR397 during fiber development of cotton [14]. Cagirici et al. reported that a stress-responsive lncRNA was the precursor of miR1117 and miR1127a [15]. In the present study, 63 lncRNAs were identified as putative precursors of 35 miRNAs, of which five miRNAs (gra-miR8753, gma-miR160b, ghr-miR7506, ghr-miR7511, and gra-miR8638) showed an expression level consistent with that of the precursor lncRNAs. For example, gma-miR160b, derived from *TCONS_00807084*, was up-regulated in the A and B lines compared with the R line. While miRNA gma-miR160b regulates an auxin response factor, *GhARF17* (*Gh_D06G0360*), was down-regulated in the A line compared with the B and R lines. Several studies indicate that miR160 and *ARF17* perform critical roles during pollen development. For example, overexpress miR160-resistant *ARF17* show male sterility in Arabidopsis [36]. Jun et al. observed that *ARFA17* is essential for primexine formation and that primexine was defective in the *arf17* mutant, which caused pollen wall-patterning defects and pollen degradation in Arabidopsis [37]. Ding et al. overexpressed miR160 in cotton, which leads to anther indehiscence, suppression of *ARF10* and *ARF17* expression, and thus increased cotton sensitivity to high temperature stress by activation of the auxin response [38]. Huang et al. increased *ARF17* expression levels by overexpressing the lncRNA bra-eTM160 for inhibition of bra-miR160 and caused male sterility in *Brassica* and observed that potential dosage-dependent regulation may render lncRNAs as an endogenous modulator for miRNA functions [19]. The above-mentioned results indicate that the lncRNAs–miR160–*ARF17* regulatory network might participate in pollen development through involvement in auxin regulation, although the underlying regulatory mechanisms are incompletely understood.

Several studies indicate that lncRNAs and miRNAs are involved in complex regulatory pathways during plant development processes [15,35,39,40]. For example, Reina et al. observed that more than 700 lncRNAs in rice were cleaved by miR2118 and processed by the DCL4 protein, resulting in production of phasiRNAs [39]. Furthermore, the lncRNA *PMS1T* is targeted by miR2118 to produce phasiRNAs that preferentially accumulate in a photoperiod-sensitive male sterility line under long-day conditions, and the elevated phasiRNAs eventually cause male sterility in rice through an unknown regulatory network [41]. Liu et al. reported a regulatory network of miR3954–lncRNA–phasiRNAs–*NAC*, which causes early flowering in citrus. Overexpression of miR3954 causes down-regulation of the corresponding lncRNAs (*Cs1g09600* and *Cs1g09635*), up-regulation of phasiRNAs, and a reduced expression level of *NAC* genes [42]. These results indicate that miRNA regulation of lncRNAs may function as part of a complex regulatory pathway during plant development. Thus, in the present study, we constructed several putative miRNAs–lncRNAs–mRNAs regulatory networks involved in CMS and fertility restoration. Fifty-eight lncRNAs regulated by 15 miRNAs and 77 lncRNAs regulated by 35 miRNAs were specifically differentially expressed in the A–B and A–R comparisons, respectively. Several miRNAs–lncRNAs–mRNAs regulatory networks were validated by qRT-PCR analysis. For example, in the A–B comparison, a transcription factor AMS gene (*Gh_D12G0328*) and a fatty acid biosynthesis-related gene (*Gh_A06G1391*), regulated by the corresponding miRNAs and lncRNAs, were down-regulated in the A line compared with the B line. These genes might be involved in CMS during anther development. In the A–R comparison, a glutamyl-tRNA reductase (Gh_A08G0634) and a calcium-dependent lipid-binding (CaLB domain) protein (Gh_D11G3015), regulated by the corresponding miRNAs and lncRNAs, function in oxidation–reduction processes and the calcium signaling pathway, respectively. An additional regulatory network, in which *PPR* (*Gh_D05G3392*) located near the *Rf1* gene mapping region is regulated by gra-miR7505b and *TCONS_00797453*, might play a critical role in fertility restoration. However, these regulatory networks require validation in the future.

## 4. Materials and Methods

### 4.1. Plant Materials and Transcriptome Sequence

The CMS-D2 three-line hybrid cotton system was developed at the Cotton Research Institute, Chinese Academy of Agricultural Science (Anyang, China). In our previous study, the CMS line harboring CMS-D2 cytoplasm was crossed with the restorer line, and the maintainer line with normal fertile Upland cotton (AD1) cytoplasm as the recurrent male parent to backcross with the F_1_ plants to construct a BC_8_F_1_ population. From this segregating population, the sterile and fertile plants were selected as the CMS-D2 line (A) and restorer line (R), respectively [27]. The A line is homozygous for the recessive (i.e., nonfunctional) fertility restorer alleles (*rf1rf1*), whereas the maintainer line (B) harbors normal fertile Upland cotton cytoplasm and has the same nuclear allelic composition (*rf1rf1*). The R line is homozygous for dominant (i.e., functional) fertility restorer alleles (*Rf1Rf1*) to allow recovery of fertility in CMS-D2 cotton plants in the cross A × R. The three lines were grown under normal production conditions. For sample collection, as described in previous studies [43,44], each genotype was grown side-by-side in field, and floral buds approximately 3 mm in length (corresponding roughly to the stage of male meiosis) were collected from about 100 plants (one floral bud was collected per plant) and combined, with three independent biological replicates. All collected floral buds were cut above the ovaries, immediately frozen in liquid nitrogen, and stored at −80 °C until use.

Total RNA was extracted using the Spectrum™ Plant Total RNA Kit (Princeton, NJ, USA) in accordance with the manufacturer’s instructions. Equal amounts of RNA from the three biological replicates were used to construct transcriptome libraries (A1–3, B1–3, and R1–3) and small RNA libraries (A, B, and R) [25]. Both transcriptome and small RNA sequencing were performed on an Illumina HiSeq 2500/2000 platform (Tianjin, China). The raw sequence data for the transcriptome and small RNAs have been submitted to the National Center for Biotechnology Information (NCBI) Sequence Read Archive (http://www.ncbi.nlm.nih.gov/sra) under accession numbers SRX3421007 and SRX3422274, respectively.

### 4.2. Annotation of Transcripts and Identification of Long Noncoding RNAs

For the transcriptome data, after filtering of low-quality reads and trimming the adaptor sequences, a total of 887,090,420 clean reads were obtained to map to the Upland cotton “Texas Marker-1” (TM-1) reference genome (http://www.cottongen.org) [45] using TopHat 2.1.1 (Seattle, WA, USA) [46]. Transcripts assembly was accomplished using Cufflinks 2.2.1 (Berkeley, CA, USA) [47] based on the results of alignment to the TM-1 reference genome. All transcripts without strand information were discarded. The remaining transcripts were used to identify lncRNAs on the basis of the following rigorous criteria. First, single-exon transcripts located within 500 bp of other transcripts were excluded. Second, transcripts smaller than 200 bp in length were removed. Third, transcripts with a fragments per kilobase of transcript per million mapped reads (FPKM) score higher than 2 with a single exon or 0.5 with multiple exons in at least one sample were retained. Fourth, transcripts that overlapped with known genes and other non-mRNAs (rRNA, tRNA, snRNA, snoRNA, and pseudogenes) were excluded. Fifth, CPC [48] and Pfamscan [49] software were used to calculate the coding potential of the remaining transcripts. Only transcripts with CPC score < 0 and Pfamscan e-value < 0.001 were considered as putative lncRNAs for subsequent analysis.

### 4.3. Expression and Target Gene Analysis of LncRNAs 

Cuffdiff 2.1.1 software was used to estimate fragments per kilobase of exon per million fragments mapped (FPKMs) of both lncRNAs and coding genes in each sample [50]. 

Based on the genome location of the lncRNAs and coding genes, we identified the target genes 10 kb upstream and downstream of lncRNAs and then analyzed their function. Gene ontology (GO) enrichment analysis was performed using the GOseq R package (version 3.6, Melbourne, Australia). The GO terms with a corrected *p*-value less than 0.05 were considered to be significantly enriched [51].

### 4.4. Prediction of Putative Targets and Endogenous Target Mimics for MiRNAs

The miRNA targets of lncRNAs or mRNAs were predicted using the psRNATarget server (http://plantgrn.noble.org/psRNATarget/), for which less than three mismatches in targets and miRNA pairing regions were permitted. The eTMs for miRNAs were predicted by combing psRNATarget and application of the rules established by Wu et al [18].

The miRNAs precursors were predicted by mapping the mature miRNAs sequence to the putative lncRNAs sequence through local BLAST and mismatch not allowed. We then utilized Mfold (http://unafold.rna.albany.edu/?q=mfold) to predict stem-loop structures as suggested by Jones-Rhoades [52].

### 4.5. Construction of MiRNA-lncRNA-mRNA Regulatory Networks

To understand the role of lncRNAs, miRNA–lncRNA–mRNA networks were constructed based on differentially expressed lncRNAs and miRNAs, and the target pairs of miRNAs–lncRNAs, miRNAs–mRNAs, and lncRNAs–mRNAs. The regulatory networks contained miRNAs, lncRNAs acting as miRNA targets, mRNAs acting as lncRNA targets, and mRNAs acting as miRNA targets. The miRNA–lncRNA–mRNA regulatory networks were visualized using Cytoscape 3.7.1 software [53]. 

### 4.6. Quantitative RT-PCR Validation of LncRNAs, MiRNAs, and mRNAs Expression

To validate the relative expression of lncRNAs, miRNAs, and mRNAs, quantitative reverse transcription PCR (qRT-PCR) was performed with specific primers as described previously [25]. Total RNAs and miRNAs were extracted from the same samples by RNA-seq and reverse-transcribed to cDNA using the TransScript^®^ miRNA First-Strand cDNA Synthesis SuperMix kit (TransGen, Beijing, China) and PrimeScript™ RT reagent kit (Takara, Dalian, China) following the manufacturers’ guidelines. The qRT-PCR mixture contained 1 μL diluted cDNA, 10 μL 2× SYBR^®^ Green Mix (Takara), 0.5 μM of each primer, and ddH_2_O to make up the volume to 20 µL. All reactions were performed with three biological replicates. *Ubiquitin 6* (*GhUBQ6*) was used as the reference gene, and relative expression levels were calculated using the 2^−ΔΔ*C*t^ method [54]. The qRT-PCR primers used are listed in Table S6.

## 5. Conclusions

In this study, systematic transcriptome sequencing was performed during anther development of Upland cotton harboring the cytoplasmic male sterile *Gossypium harknessii* (D2) cytoplasm. In total, 80,695 lncRNAs were identified, of which 43,347 and 44,739 lncRNAs were differentially expressed in the A–B and A–R comparisons, respectively. These lncRNAs represent functional candidates involved in CMS and fertility restoration. We analyzed the putative relationship between lncRNAs and miRNAs and observed that lncRNAs may act as miRNA precursors, miRNA targets, and miRNA eTMs. Sixty-three lncRNAs were identified as putative precursors of 35 miRNAs, and qRT-PCR results showed a similar expression level to that of RNA-seq data. To explore the functions of lncRNAs, we constructed putative miRNA–lncRNA–mRNA regulatory networks involved in CMS and fertility restoration. However, further functional analyses are needed to elucidate the regulatory networks. This study lays a solid foundation for exploration of the functions and regulatory mechanisms of lncRNAs in anther development of cotton.

## Figures and Tables

**Table 1:** Summary of the data of transcriptome in A, B, and R lines.

	A1	A2	A3	B1	B2	B3	R1	R2	R3
Raw Reads	81,937,808	91,168,084	121,573,148	110,284,226	100,663,168	104,176,106	100,740,046	105,585,106	104,618,514
Clean Reads	78,996,350	87,941,972	116,946,292	106,115,854	96,899,546	100,304,158	97,149,736	101,878,248	1008,58,264
Total Mapped Reads	69,087,555	76,275,950	101,026,262	92,535,847	84,731,402	87,778,855	85,862,554	90,725,753	89,536,452
Mapped Unique Reads	62,121,536	67,953,623	89,895,211	82,087,734	75,065,210	77,755,238	76,292,918	81,060,568	79,962,304
Overall Mapping	87.46%	86.73%	86.39%	87.20%	87.44%	87.51%	88.38%	89.05%	88.77%
Reads Mapped to mRNA	21,490,343	24,321,071	31,839,943	29,301,509	27,050,077	27,548,951	27,405,358	29,115,198	28,012,425
Total lncRNA	80,695								

Columns represent: A1–A3: the CMS line with three biological replicates; B1–B3: maintainer line with three biological replicates; R1–R3: fertility restoration line with three biological replicates.

## Supplementary Materials

The following are available online at https://www.mdpi.com/1422-0067/20/22/5530/s1.

## Abbreviations

CMS	Cytoplasmic male sterility
Rf gene	Restorer-of-fertility gene
LncRNAs	Long noncoding RNAs
A	CMS line
B	Maintainer line
R	Restorer-of-fertility line
GO	Gene ontology
FPKM	Fragments per kilobase of exon per million fragments
